# Global disparities in cancer supportive care: An international survey

**DOI:** 10.1002/cam4.70234

**Published:** 2024-09-13

**Authors:** Alexandre Chan, Lawson Eng, Changchuan Jiang, Mary Dagsi, Yu Ke, Mary Tanay, Cristiane Bergerot, Niharika Dixit, Ana Cardeña Gutiérrez, Ana I. Velazquez, Farhad Islami, Enrique Soto‐Perez‐de‐Celis

**Affiliations:** ^1^ School of Pharmacy & Pharmaceutical Sciences University of California Irvine Irvine USA; ^2^ National Cancer Centre Singapore Singapore Singapore; ^3^ Division of Medical Oncology and Hematology, Department of Medicine Princess Margaret Cancer Centre/University Health Network, University of Toronto Toronto Ontario Canada; ^4^ Division of Hematology and Oncology, Department of Internal Medicine University of Texas Southwestern Medical Center Dallas Texas USA; ^5^ Florence Nightingale Faculty of Nursing, Midwifery and Palliative Care, King's College London London UK; ^6^ Oncoclinicas Brasilia DF Brazil; ^7^ University of California, San Francisco/Zuckerberg San Francisco General Hospital San Francisco California USA; ^8^ Medical Oncology Department Hospital Universitario Nuestra Señora de Candelaria Santa Cruz de Tenerife Spain; ^9^ University of California, San Francisco, Helen Diller Family Comprehensive Cancer Center San Francisco California USA; ^10^ Surveillance and Health Equity Science, American Cancer Society Atlanta Georgia USA; ^11^ Department of Geriatrics Instituto Nacional de Ciencias Médicas y Nutrición Salvador Zubirán Mexico City Mexico; ^12^ Division of Medical Oncology University of Colorado Anschutz Medical Campus Aurora Colorado USA

**Keywords:** cancer supportive care, financial toxicities, health disparities, healthcare professionals, low‐ and middle‐income countries, social needs

## Abstract

**Background:**

The global cancer burden is rising, particularly in low‐ and middle‐income countries (LMIC), highlighting a critical research gap in understanding disparities in supportive care access. To address this, the Multinational Association of Supportive Care in Cancer (MASCC) Health Disparities Committee initiated a global survey to investigate and delineate these disparities. This study aims to explore and compare supportive care access disparities between LMIC and High‐Income Countries (HIC).

**Methods:**

An online cross‐sectional survey was conducted among active members of MASCC. Members, representing diverse healthcare professions received email invitations. The survey, available for 3 weeks, comprised sections covering (1) sociodemographic information; (2) clinical service/practice‐related disparities in their region/nation; (3) population groups facing disparities within their region or country. Chi‐squared or Fisher's exact test for cross‐sectional analyses, and a multivariable logistic regression model was employed for statistical analysis.

**Results:**

A total of 218 active members participated, with one‐quarter (26.6%) from LMIC and 18.4% ethnic minorities, timely cancer care (43.7%) and timely supportive care (45.0%) emerged as the most pressing disparities globally. Notably, participants from LMIC underscored cancer drug affordability (56.4%) and supportive care guideline implementation (56.4%) as critical issues. Economically disadvantaged populations were noted as more likely to face disparities by both LMIC and HIC (non‐US‐based) respondents, while US‐based respondents identified racial/ethnic minorities as facing more disparities.

**Conclusion:**

This global survey reveals significant disparities in cancer supportive care between LMIC and HIC, with a particular emphasis on medication affordability and guideline implementation in LMIC. Addressing these disparities requires targeted intervention, considering specific regional priorities.

## 1 INTRODUCTION

Despite significant advancements in cancer screening, diagnosis, and treatment, the global burden of cancer continues to grow significantly. According to the Global Burden of Disease Study 2019, there are 23.6 million new cancer cases worldwide, leading to 10 million deaths and an estimated 250 million disability‐adjusted life years attributed to cancer. Alarmingly, low‐ and middle‐income countries (LMIC) have experienced more pronounced increases in cancer burden.^1^

Supportive care, defined as the care that helps a person and their family cope with the effects of both cancer and its treatment, fills the gap between cancer‐directed treatment and the daily lives of patients and their families.^2^ Numerous studies have demonstrated that supportive care enhances the quality of life for patients with cancer through effective pain management, emotional support, and nutritional guidance, among others.^3^ Moreover, the integration of supportive care into cancer care has been linked to decreased utilization of acute care services, reduced medical costs, and often improved patient outcomes.^4^, ^5^

Despite the potential benefits of integrating supportive care into routine cancer care, there remains significant heterogeneity in its integration and access across patient populations. This is similar to other areas of cancer care where there are well‐recognized disparities. In a recent American Cancer Society report on cancer disparities in the United States, there was significant variability in screening rates, mortality, and survival between sociodemographic groups and by race and ethnicity.^6^ Much of this variability is attributed to differences in exposure to risk factors, access to screening, and receipt of treatment (including guidance concordant treatment). Factors that can impact the receipt of treatment (including appropriate supportive care) include access to insurance and provider expertise, treatment costs, and geographic (rural vs. urban) differences. There are similar disparities in access to supportive and/or end‐of‐life care with variability seen between LMIC and high‐income countries (HIC) and within the United States and other HIC.^7^, ^8^, ^9^, ^10^ Some factors influencing access to palliative and supportive care include race/ethnicity group, place of birth, geographical region (urban/rural), cultural beliefs around supportive/palliative care, and demographic factors (age, gender, income).^11^ On a more global level, some of the factors that may lead to disparities seen in supportive care across different regions of the world include access to cancer/supportive care medicines, political/government environments, differences in the training of healthcare personnel and workforce, and the varying socio‐demographics and clinical presentations of patients with cancer.^12^, ^13^ Although previous research addresses disparities in cancer supportive care on a global level, there is limited understanding in the different disparities that individuals may face in their particular region. Many factors can contribute to this including but not limited to societal, economic, and political factors. This study aims to fill these gaps by gathering input from healthcare professionals around the world to identify region‐specific issues and systemic factors that may contribute to the different disparities that patients and healthcare providers face.

Although the global cancer burden continues to rise, with a particularly rapid increase in LMIC, there is a lack of research focusing on disparities in access to supportive care for patients with cancer. Recognizing this gap, the Multinational Association of Supportive Care in Cancer (MASCC) Health Disparities Committee conducted a global survey on this issue. We hypothesized that LMICs experience different supportive care barriers compared to HIC. As the largest international, multidisciplinary organization devoted to supportive care in cancer, MASCC can reach out to multidisciplinary cancer physicians, nurses, pharmacists, and other healthcare team members across the world. The findings from this global survey are expected to inform various stakeholders on resource allocation and intervention strategies and ultimately improve patient outcomes across diverse socioeconomic settings.

## 2 METHODS

### 2.1 Study design

This is a cross‐sectional survey administered to active members of the MASCC, a global society of healthcare professionals and researchers in cancer supportive care. This study was exempted by the University of California, Irvine Investigational Review Board (IRB), and a waiver of informed consent was granted by the IRB.

### 2.2 Inclusion/exclusion criteria

All active MASCC members in March 2023 were eligible and received an invitation to the survey via email. At the time of survey dissemination, MASCC had 2137 members in 70 countries. Members of MASCC, which include physicians, nurses, dental professionals, pharmacists, dieticians, physiotherapists, psychologists, and other healthcare professionals are required to undergo verification when they join the organization.

### 2.3 Data collection

Members of the MASCC Health Disparities Task Force drafted the survey questions before they were assessed by the MASCC Executive Committee for appropriateness before circulation by task force members and by the MASCC Board of Directors for content validity. The survey questionnaire was built electronically using SurveyMonkey. The electronic survey was disseminated using MASCC membership email list in March 2023, with three weekly email reminders. The survey was available to responders for 3 weeks.

The survey was divided into three sections. The first section consisted of 12 sociodemographic questions related to the membership. Following these questions, participants were asked to rate the clinical service/practice‐related disparities in their region/nation using a 1–5 Likert scale, with 5 representing the highest importance and 1 representing the least importance. Services and practices included: social service access, cancer drug affordability, supportive care drug affordability, availability of supportive care guidelines in the local context, implementation of supportive care guidelines, availability of evidence‐based cancer care guidelines in the local context, and availability of timely cancer care and timely supportive care.

Lastly, participants were asked to rank population groups facing disparities within their region or country from 1 to 6, with 6 representing the population group that they considered to be the most important priority and 1 representing the population group that they considered to be the least important priority. The following populations were included: rural populations, pediatric and adolescent/young adult (AYA) patients, older adults, LGBTQ+, race/ethnic minorities (including indigenous health), and economically disadvantaged populations (living in poverty).

### 2.4 Analysis

We first described the proportion of respondents reporting clinical service/practice‐related disparities in their region/nation, which were stratified based on respondents' characteristics (HIC vs. LMIC, self‐reported as minority, gender, and profession). Then, we evaluated whether respondents from LMIC are more likely to strongly agree that they face health disparities in specific services/practices. Lastly, we compared population groups that are more likely to face disparities within their region or country, stratified based on respondents' characteristics (HIC vs. LMIC, self‐reported as minority, gender, and profession).

### 2.5 Statistical analysis

Descriptive statistics was used to summarize responses to each item. Categorical data were presented as counts and percentages. Chi‐squared test or Fisher's exact test was conducted for cross‐sectional analyses to evaluate whether participants from LMIC agreed more strongly with the presence of disparities in specific areas. This was followed by a multivariable logistic regression model performed with adjustments for ethnic minority status, gender, profession, age, and years of practice experience. Odds ratios (with 95% confidence interval) were used to present the effect size of the association. A 95% confidence interval that excluded unity and two‐sided *p* < 0.05 was considered significant. All statistical analysis was conducted using SPSS version 28.

## 3 RESULTS

### 3.1 Demographics

The survey was sent to 2137 MASCC members and a total of 218 members responded. The overall response rate was 10.2%. The majority (73.4%) of participants are from HIC, and 18.4% self‐identified as minorities. (Table 1) The majority were female (56.9%) and between the ages of 41–60 years (56.5%). 55.1% of the participants were physicians and 15.2% were nurses, 76.2% had been working more than 10 years in their respective fields, and 60.6% worked in the public sector. In terms of involvement with MASCC, more than half reported being members for less than 3 years (51.7%). The demographics of respondents were similar when compared between HIC and LMIC, except age (*p* = 0.05) and years of practice (*p* = 0.02). (Table S1).

**TABLE 1:** Demographic information (*n* = 218).

Demographics	*N* (%)
Age (years)	
21–30	7 (3.2%)
31–40	54 (24.8%)
41–50	64 (29.4%)
51–60	59 (27.1%)
61–70	30 (13.8%)
71–80	4 (1.8%)
Gender	
Male	94 (43.1%)
Female	124 (56.9%)
Countries	
Low‐ and Middle‐Income Country	56 (25.7%)
High‐Income Country	160 (73.4%)
Missing	2 (0.9%)
Self‐identified as minority	
Yes	40 (18.4%)
Current professional role	
Physician	120 (55.1%)
Nurse	33 (15.2%)
Pharmacist	10 (4.6%)
Dentist/oral surgeon	8 (3.7%)
Trainees/student	4 (1.8%)
Psychologist	3 (1.4%)
Physiotherapist	3 (1.4%)
Others (e.g., researchers, dietitian)	36 (16.6%)
Years worked in respective field	
<1 year	3 (1.4%)
1–5 years	22 (10.1%)
6–10 years	27 (12.4%)
>10 years	166 (76.2%)
Current practice	
Public sector	129 (60.6%)
Private sector	43 (20.2%)
Both	31 (14.6%)
Other	10 (4.7%)
Duration as member of MASCC	
< 1 year	46 (21.6%)
1–3 years	64 (30.1%)
4–5 years	27 (12.7%)
6–10 years	47 (22.1%)
>10 years	29 (13.6%)

### 3.2 Importance of specific disparities

When asked which cancer‐related disparities were of the highest importance, timely cancer care (43.75%) and timely supportive care (45%) were ranked as the most important overall.

Participants from HIC and the United States ranked timely cancer care (43.6–46.2%) and timely supportive care (41.9%–48.7%) as the most important disparities in their country or region. In contrast, participants from LMIC reported timely cancer care (59.0%) and both cancer drug affordability (56.4%) and implementation of supportive care guidelines (56.4%) as the most important disparities in their country or region. (Table 2) Technology utilization (20.5%–23.1%) and availability of supportive care guidelines in the local context (12.8%–22.2%) were least agreed as disparities in HIC and the United States, whereas access to social services (15.4%) was least agreed as a disparity in LMIC.

**TABLE 2:** Clinical service and/or practice‐related health disparities faced by respondents in their region/nation.

	Social Services Access	Cancer Drug Affordability	Supportive Care Drug Affordability	Availability of supportive care guideline in local context	Implementation of Supportive Care Guideline	Availability of Evidence‐Based Cancer Care Guideline in Local Context	Timely Cancer Care	Timely Supportive Care	Technology Utilization
Respondent characteristics									
Income group									
Low‐Middle Income	15.4%	56.4%	43.6%	38.5%	56.4%	41.0%	48.7%	59.0%	35.9%
High Income–others	18.8%	33.3%	26.5%	22.2%	27.4%	23.9%	43.6%	41.9%	20.5%
High Income–USA	23.1%	38.5%	20.5%	12.8%	17.9%	23.1%	48.7%	46.2%	23.1%
Self‐identified as minority									
Minority	13.3%	33.3%	30.0%	30.0%	33.3%	30.0%	40.0%	40.0%	22.7%
Non‐Minority	18.8%	39.8%	30.5%	25.8%	34.4%	28.1%	46.1%	47.7%	30.0%
Gender									
Male	19.7%	38.0%	26.8%	23.9%	32.4%	23.9%	36.6%	39.4%	26.8%
Female	16.5%	40.0%	34.1%	28.2%	36.5%	31.8%	51.8%	51.8%	22.4%
Profession									
Physician	17.2%	36.6%	28.0%	25.8%	34.4%	24.7%	40.9%	44.1%	23.7%
non‐Physician	18.8%	42.2%	27.5%	34.4%	34.4%	32.8%	50.0%	48.4%	25.0%

Participants who self‐identified as a minority reported timely cancer care (40.0%) and timely supportive care (40.0%) as their top two of the highest importance. Similarly, participants who self‐identified as non‐minority ranked timely cancer care (46.1%) and timely supportive care (47.7%) as most important.

Specific disparities were identified by respondents from LMIC (Table 3). Compared to responses from HICs, respondents from LMIC were at higher odds to strongly agree that there were disparities in cancer drug affordability (OR = 3.71, 95%CI:1.68–8.51), supportive care drug affordability (OR = 2.76, 95%CI: 1.21–6.32), availability of supportive care guideline in local context (OR = 2.64, 95%CI: 1.11–6.28), implementation of supportive care guideline in local context (OR = 3.91, 95%CI: 1.74–8.81), availability of evidence‐based cancer care guideline in local context (OR = 2.27, 95%CI: 1.01–5.10), and timely supportive care (OR = 2.43, 95%CI: 1.09–5.40) in the country or region that they reside in.

**TABLE 3:** Agreement with services/practices health disparities faced by respondents from low‐middle income countries, compared to respondents from high income countries.

Strong Agreement with Health Disparities	Odds Ratios (95%CI), *p* value	Adjusted odd ratios* (95%CI), *p* value
Cancer Drug Affordability	2.62 (1.25–5.51), *p* = 0.011	3.71 (1.68–8.51), *p* < 0.001
Supportive Care Drug Affordability	2.19 (1.03–4.67), *p* = 0.04	2.76 (1.21–6.32), *p* < 0.016
Availability of Supportive Care Guideline in Local Context	2.25 (1.03–4.92), *p* = 0.042	2.64 (1.11–6.28), *p* = 0.0284
Implementation of Supportive Care Guideline	3.37 (1.72–7.82), p < 0.001	3.91 (1.74–8.81), p < 0.001
Availability of Evidence‐Based Cancer Care Guideline in Local Context	2.27 (1.05–4.90), *p* = 0.037	2.27 (1.01–5.10), *p* = 0.0476
Timely Supportive Care	2.08 (0.99–4.35), *p* = 0.052	2.43 (1.09–5.40), *p* = 0.0298

* Adjusted for ethnic minority status, gender, profession, age and years of practice experience.

### 3.3 Population groups facing disparities

Participants from HIC other than the United States ranked the following population groups in the following order: economically disadvantaged (14.4%), rural populations (13.1%), race/ethnic minorities (11.2%), geriatrics (8.1%), in pediatric/AYA (5.6%), and LGBTQ+ cancer care (2.5%). (Table 4). Participants from the United States, however, ranked disparities in race/ethnic minorities (22.9%) first, followed by economically disadvantaged (16.7%), rural populations (14.6%), geriatrics (8.3%), pediatric/AYA (2.1%), and LGBTQ+ (2.1%). Similar to participants from HIC other than the United States, participants from LMIC ranked economically disadvantaged first at a substantially greater proportion (32.1%), followed by pediatric/AYA (10.7%), LGBTQ+ (5.4%), geriatric (3.6%), and rural populations (3.6%). In contrast to participants from HIC and the United States, however, concern about disparities in race was minimal among participants from LMIC (0.0%).

**TABLE 4:** Respondents' opinion on population groups that are mostly likely to face supportive care disparities.

	Population Groups					
	Rural Populations	Pediatric/AYA	Geriatrics	LGBTQ+	Race/ethnic minorities (including indigenous groups)	Economically disadvantaged populations
Respondent Characteristics						
Income Group						
Low‐Middle Income	3.6%	10.7%	3.6%	5.4%	0%	32.1%
High income–Others	13.1%	5.6%	8.1%	2.5%	11.2%	14.4%
High income–USA	14.6%	2.1%	8.3%	2.1%	22.9%	16.7%
Self‐identified as minority						
Minority	7.5%	2.5%	2.5%	7.5%	5.0%	25.0%
Non‐minority	11.6%	7.7%	7.7%	2.2%	8.8%	17.1%
Gender						
Male	11.8%	7.5%	5.4%	3.2%	6.5%	20.4%
Female	10.5%	6.5%	8.1%	3.2%	9.7%	17.7%
Profession						
Physician	11.7%	7.5%	6.7%	2.5%	6.7%	24.2%
Non‐physician	10.2%	6.1%	7.1%	4.1%	10.2%	12.2%

In terms of other respondent characteristics which include self‐reported ethnic minority status, gender, and profession, all respondents ranked economically disadvantaged populations as the top population group of concern, with rural populations ranking second. Non‐physicians also ranked racial/ethnic minorities second for disparity concerns.

## 4 DISCUSSION

This study evaluates the global perspective on health disparities that are commonly faced by cancer care providers who are interested in cancer supportive care. Interestingly, the most important disparities reported by respondents from HIC were mostly related to timely cancer care and timely supportive care, whereas those from LMIC were mostly related to timely cancer care, cancer drug affordability, and implementation of care guidelines. Aligning with our hypothesis, respondents from LMIC were more agreeable than respondents from HIC that disparities existed in cancer drug affordability, supportive care drug affordability, availability of supportive care guidelines in local contexts, implementation of supportive care guidelines in local contexts, and timely supportive care in their country or region than respondents from HIC. These responses highlight that access to timely care is still a problem within the supportive care community. Despite global efforts to publish and disseminate supportive care guidelines, their implementation in LMIC remains challenging due to issues such as lack of applicability and/or financial barriers.

There were also differences between respondents from LMIC and HIC regarding the ranking of their priorities in reducing disparities in cancer care. Respondents from LMIC were more focused on cancer and age‐specific subpopulations including older adults and AYA/pediatric cancers. This may be due to the limited availability of providers and resources for these sub‐specialties.^14^, ^15^ Specifically for geriatric oncology, challenges include the availability of trained personnel in geriatric oncology, lack of knowledge and tools to perform a geriatric assessment, and lack of time to perform a geriatric assessment during clinical encounters. Other challenges for providing geriatric oncology care in LMIC include limited geriatric oncology fellowship programs available in LMIC, which may contribute to the lack of available providers in this area.^16^ Furthermore, the infrastructure for geriatric assessments in LMIC might be challenging as geriatricians may not be integrated into the same practice site or campus as cancer care, leading to patients having to travel and spend further out‐of‐ pocket costs for geriatric care.^14^ This is becoming particularly important given the increasing population growth, overall life expectancy, and improvements in the early detection of cancer and treatment options,^17^ which likely contributed to its importance in HIC.

Common to both LMIC and HIC are reducing disparities between socio‐economic groups. However, the priority unique to HIC is rural–urban disparities and racial disparities. In particular, double of the respondents from the United States recognizes racial/ethnic minorities as a group at risk for disparities compared to other HIC, compared to no respondents from LMIC recognizing this issue as a top disparity issue, suggesting that racial disparity is likely more prevalent in countries with a history of colonization, slavery, and segregation of minoritized groups. Interestingly, as we evaluated respondent characteristics that may influence their perspectives on health disparities, we observed that timeliness of both cancer care and supportive care are reported as the top two disparities observed in their country/region among self‐reported minorities. Putting together, disparities in cancer care extend beyond access to cancer supportive care to disparities in access to screening, cancer treatment, outcomes, and differences in the quality of cancer care delivered.^18^, ^19^, ^20^, ^21^, ^22^, ^23^, ^24^ This difference in priority specific to HICs is likely due to the heterogeneous availability and access to cancer care in specific regions within HIC, while in LMIC availability and access remain a challenge in many regions.

Similar to providing cancer care for older adults, pediatric cancer care is an important priority in LMICs. Over time, the leading cause of mortality in young children in LMIC has shifted from communicable diseases towards non‐communicable diseases where cancer is becoming one of the leading causes of mortality.^25^ Treatment of pediatric cancers requires specialized personnel (i.e., oncologists, pathologists, radiation oncologists, surgeons, nurses, pharmacists, allied health specialists) in pediatric oncology and the appropriate infrastructure (inpatient beds/intensive care facilities, laboratory, blood products/medications, and surgical/radiation facilities) to deliver care^15^; which may be limited in resource‐limited settings. This lack of personnel and infrastructure can potentially delay access to care in these LMIC settings.^26^ Furthermore, a recent systematic review has shown that treatment‐related mortality was higher in children with cancer from LMIC when compared to HIC.^27^ Additionally, the mortality rates in HIC have been down‐trending over time while mortality rates have remained stable in LMIC. This disparity could be explained by limited supportive care interventions available for treatment‐related toxicities, further emphasizing why this group of patients was prioritized in LMIC.

To address these global disparities effectively, we propose utilizing a structured approach to integrate research findings into practice by emphasizing tailored dissemination strategies that account for regional contexts and barriers. For LMICs, it involves developing locally applicable guidelines and overcoming financial and logistical challenges. For example, the Practical, Robust Implementation and Sustainability Model (PRISM) framework highlights the importance of ensuring long‐term sustainability through continuous evaluation, stakeholder engagement, and resource allocation.^28^ Many similar frameworks are available, and they can be used to advocate practical, actionable steps, such as creating training programs, forming local partnerships, and setting up monitoring mechanisms to track the progress of guideline implementation. Few studies collect such outcomes in LMIC with the success of guidelines implementation, and we would like to encourage researchers to publish their implementation experiences using these metrics.

Given the complex and often overwhelming nature of these disparities, healthcare systems worldwide must take decisive actions to improve supportive care. Achieving universal health coverage (UHC) for patients with cancer and other conditions causing serious health‐related suffering (SHS) is a vital goal, and supportive care interventions must align with the World Health Organization's Essential Medicines Lhhist while being adaptable to various settings. By implementing the PRISM framework, we can make significant strides towards this goal.

While this objective may appear ambitious, there are various ways in which researchers, advocates, and policymakers can contribute to progress towards it. In contrast with other interventions, supportive care represents a relatively affordable and high‐value investment that can provide large returns and lead to cost savings by reducing admissions near the end of life, and research into the cost‐effectiveness of supportive care in various scenarios should be undertaken.^29^ Another potential first step which is within the reach of international organizations such as MASCC and is recommended by the Lancet Commission on Palliative Care, is creating and implementing an affordable and cost‐effective essential package of palliative and supportive care interventions that can be easily deployed across various settings in HIC and LMIC.^30^

**FIGURE 1:**
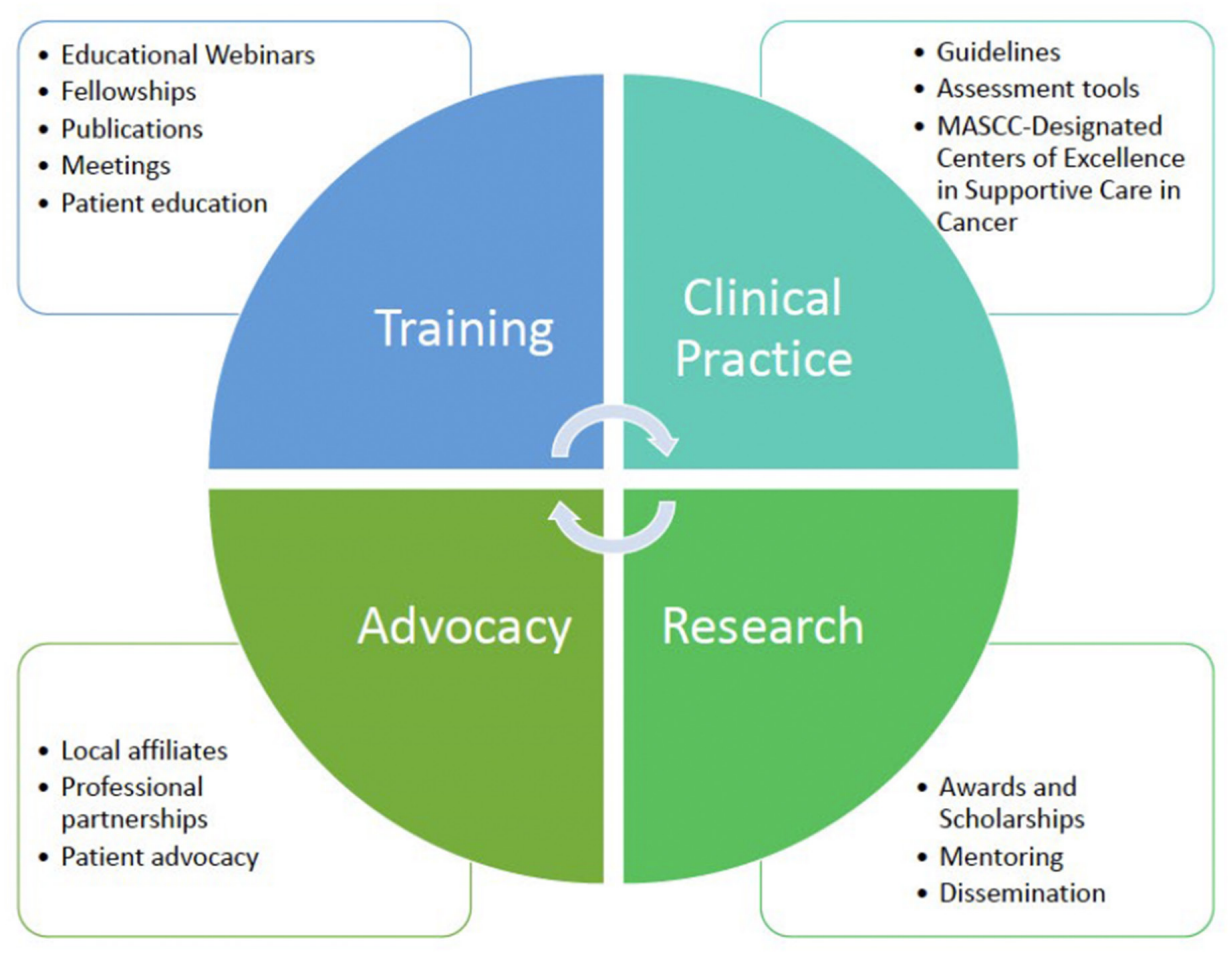
Roles that MASCC can play to reduce health disparities in both HIC and LMIC.

Global organizations like MASCC have a crucial role in advocating UHC in both HIC and LMIC through various programs and initiatives that include training, clinical practice support, research, and advocacy (Figure 1). The multidisciplinary nature of global organizations can allow the creation of training modules and courses that can be utilized to increase the supportive care competencies of the entire workforce, further empowering “generalist” supportive care and task‐shifting (i.e. provision of specialized care by general practitioners, nurses, pharmacists, or community health workers) in understaffed areas. Supporting practices through the generation of models for supportive care integration into the existing healthcare system is another area in which organizations can make a difference, particularly through the creation of guidelines, such as resource‐stratification to fit various environments.^31^ Likewise, fostering an implementation research agenda studying the integration of supportive care into everyday clinical practice, particularly at the community level, in resource‐constrained areas, is another potential task that organizations can undertake, both through funding and dissemination of results in meetings and publications.^32^, ^33^ Finally, organizations have an important role to play in raising public awareness of the importance of supportive care, with a focus on disparities and barriers to accessing it in both HIC and LMIC. Organizations should also strive to obtain firsthand information from their members on the ground at the local level and meld this into global initiatives aimed at changing policies for healthcare delivery.^30^

There are a few limitations in our study. Although we have a sizable membership within our organization, we faced a relatively low response rate with this survey. However, such response rate is similar to previous surveys conducted by the organization.^34^, ^35^, ^36^ Hence, not all results are generalizable to the entire membership. Furthermore, the majority of our membership (>80%) reside in HIC within North America, Western Europe and the Western Pacific, making it a challenge to gather more responses representing LMIC where disparities tend to be more prevalent. It is also important to note that organizations such as MASCC are paid memberships and may not fully represent the diverse healthcare professionals globally, especially those from LMIC. Despite this being a global survey targeting respondents from different countries, we administered the survey in English only as it is the official language of MASCC. To improve participation of our global membership, we have employed strategies to increase the response rate through numerous reminders to invite members to participate in this survey. Furthermore, providing the survey in different languages can encourage participation and better represent other countries, especially LMIC. The study relies on self‐reported data as well and each respondent may have their subjective interpretations of the questions. This may affect the accuracy of responses. Lastly, the data in this survey provides data regarding the different disparities that existed at the time of this survey. Conducting longitudinal responses may provide a better understanding of different disparities that occur over time.

## 5 CONCLUSION

Through a global survey of our supportive care network, we have observed different trends of supportive care disparities between LMIC and HIC. In HIC, supportive care disparities are mostly identified to be in the areas of timely cancer care and timely supportive care, whereas the areas identified in LMIC are mostly related to timely cancer care, cancer drug affordability, and implementation of care guidelines. Consistently, both respondents from LMIC and HIC ranked socioeconomic status inequalities as the most important social issues faced that would lead to health disparities. MASCC and similar organizations are well‐positioned to address these disparities by advocating for UHC, providing targeted programs, and enhancing local engagement. Implementing frameworks may further support effective guideline dissemination and integration into practice, addressing key disparities and enhancing supportive care. This approach offers practical strategies for improving implementation and ensuring the sustainability of supportive care interventions.

## AUTHOR CONTRIBUTIONS

**Alexandre Chan:** Conceptualization (lead); data curation (lead); formal analysis (lead); investigation (lead); methodology (lead); project administration (lead). **Lawson Eng:** Conceptualization (equal); data curation (equal); formal analysis (equal); investigation (equal); methodology (equal). **Changchuan Jiang:** Conceptualization (equal); data curation (equal); formal analysis (equal); investigation (equal); methodology (equal). **Mary Dagsi:** Conceptualization (equal); data curation (equal); formal analysis (equal); investigation (equal); methodology (equal). **Yu Ke:** Conceptualization (equal); data curation (equal); formal analysis (equal); investigation (equal); methodology (equal). **Mary Tanay:** Conceptualization (equal); data curation (equal); formal analysis (equal); investigation (equal); methodology (equal). **Cristiane Bergerot:** Conceptualization (equal); data curation (equal); formal analysis (equal); investigation (equal); methodology (equal). **Niharika Dixit:** Conceptualization (equal); data curation (equal); formal analysis (equal); investigation (equal); methodology (equal). **Ana Cardeña Gutiérrez:** Conceptualization (equal); data curation (equal); formal analysis (equal); investigation (equal); methodology (equal). **Ana I. Velazquez:** Conceptualization (equal); data curation (equal); formal analysis (equal); investigation (equal); methodology (equal). **Farhad Islami:** Conceptualization (equal); data curation (equal); formal analysis (equal); investigation (equal); methodology (equal). **Enrique Soto‐Perez‐de‐Celis:** Conceptualization (equal); data curation (equal); formal analysis (equal); investigation (equal); methodology (equal).

## Supporting information


Table S1:

## Data Availability

The data that support the findings of this study are available based on request.
