# Supplementary material for: Global disparities in cancer supportive care: An international survey
Source: Cancer Med. 2024 Sep 13;13(17):e70234. doi: 10.1002/cam4.70234 (PMC11393557; doi:10.1002/cam4.70234)
Supplement: Supplementary file 1 — Table S1: [file CAM4-13-e70234-s001.docx]

**Supplementary Table 1: Comparison of characteristics between LMIC and HIC respondents**

| Age (years) |  |  |
| --- | --- | --- |
| 21-30 | 4 (2.5%) | 3 (5.4%) |
| 31-40 | 31 (19.4%) | 21 (37.5%) |
| 41-50 | 48 (30.0%) | 16 (28.6%) |
| 51-60 | 48 (30.0%) | 11 (19.6%) |
| 61-70 | 25 (15.6%) | 5 (8.9%) |
| 71-80 | 4 (2.5%) | 0 |
| Gender |  |  |
| Male | 68 (42.5%) | 25 (44.6%) |
| Female | 92 (57.5%) | 31 (55.4%) |
| Self-identified as minority |  |  |
| Yes | 26 (16.3%) | 14 (25.0%) |
| Current professional role |  |  |
| Physician | 85 (53.1%) | 34 (60.7%) |
| Nurse | 26 (16.3%) | 7 (12.5%) |
| Pharmacist | 6 (3.8%) | 3 (5.4%) |
| Dentist/oral surgeon | 4 (2.5%) | 4 (7.1%) |
| Trainees/student | 4 (2.5%) | 0 |
| Psychologist | 2 (1.25%) | 1 (1.8%) |
| Physiotherapist | 3 (1.9%) | 0 |
| Others (e.g., researchers, dietitian) | 29 (18.1%) | 7 (12.5%) |
| Years worked in respective field |  |  |
| < 1 year | 1 (0.63%) | 2 (3.6%) |
| 1-5 years | 13 (8.1%) | 8 (14.3%) |
| 6-10 years | 16 (10.0%) | 10 (17.9%) |
| >10 years | 130 (81.3%) | 36 (64.3%) |
| Current practice |  |  |
| Public sector | 102 (63.8%) | 26 (46.4%) |
| Private sector | 24 (15.0%) | 19 (33.9%) |
| Both | 23 (14.4%) | 8 (14.3%) |
| Other | 8 (5.0%) | 2 (3.6%) |
| Duration as member of MASCC |  |  |
| < 1 year | 30 (18.8%) | 15 (26.8%) |
| 1-3 years | 47 (29.4%) | 17 (30.4%) |
| 4-5 years | 19 (11.9%) | 8 (14.3%) |
| 6-10 years | 34 (21.3%) | 13 (23.2%) |
| >10 years | 27 (16.9%) | 2 (3.6%) |
